# Silk Microfiber-Reinforced Biomass Aerogel with Cobweb-like Pore Structure for Highly Efficient Eco-Friendly Air Filtration

**DOI:** 10.3390/gels12050443

**Published:** 2026-05-19

**Authors:** Kao Wu, Zihan Yu, Zixuan Yang, Yingjie Ding, Hong Qian, Ying Kuang, Man Xiao, Fatang Jiang, Bo Peng

**Affiliations:** 1 Cooperative Innovation Center of Industrial Fermentation (Ministry of Education & Hubei Province), School of Life and Health Sciences, Hubei University of Technology, Wuhan 430068, China; wukao@mail.hbut.edu.cn (K.W.); lazywawa@163.com (Y.K.); 20061070@hbut.edu.cn (M.X.); jiangft@mail.hbut.edu.cn (F.J.); 2 School of Nursing and Health Management, Wuhan Donghu University, Wuhan 430212, China; 3 Department of Architecture and Built Environment, Faculty of Engineering, University of Nottingham, Nottingham NG7 2RD, UK

**Keywords:** aerogel, air filtration, silk microfibers, pore structure, mechanical property

## Abstract

Airborne particulate matter pollution has posed severe threats to public health, while conventional air filtration materials suffer from non-biodegradability and poor structural stability. Herein, a series of eco-friendly konjac glucomannan/sodium alginate (KGM/SA) composite aerogels reinforced by silk microfibers (SFs) were fabricated via freeze-drying. The extracted SF had a concentrated diameter distribution of 500 nm, with a well-preserved crystalline structure and the β-sheet secondary structure of natural silk. Results demonstrated that SF incorporation effectively regulated the pore structure, with reduced pore sizes, and an optimized uniform and compact cobweb-like porous network was achieved at 70% SF addition (KSSF_70_), with a maximum compressive stress of 78.89 kPa at 60% strain, a PM_10_ filtration efficiency of 99.8%, and a PM_2.5_ efficiency of 71.2%. Also, the removal efficiency of particles < 0.3 μm was boosted from 26% to 47% compared with the KGM/SA aerogel. Furthermore, the calculated quality factor met mainstream commercial standards. These findings guided SF use in improving the pore structure of biomass aerogels for enhanced air filtration performance.

## 1. Introduction

With the developing industrialization and urbanization, atmospheric particulate matter pollution (such as PM_2.5_) and industrial waste gas emissions have become increasingly severe. These gaseous pollutants spread in the air and induce severe infectious diseases such as influenza and pneumonia [1]. At present, mainstream air purification materials (mainly polypropylene melt-blown fabrics [2], glass fibers [3], etc.) possess advantages including low cost and high productivity, yet they have a critical environmental limitation of non-biodegradability. Thus, the development of high-performance and biodegradable bio-based air filtration materials has become a research hotspot in the field of environmentally friendly materials [4,5]. Against this background, biomass-based aerogel materials have emerged as an ideal alternative to traditional materials by virtue of their unique structural and performance advantages [6,7]. Aerogels are lightweight porous materials formed by replacing the liquid phase components in wet gels with gas while retaining their three-dimensional porous network structure [8]. Their three-dimensional porous network structure, together with the characteristics of high porosity and high specific surface area, enables them to not only improve the filtration efficiency by increasing the contact probability between pollutants and the materials, but also provide low-resistance pathways for gas circulation, which meets the core requirement of high efficiency and low resistance for air filtration materials [9]. In particular, biomass-based aerogels based on natural polysaccharides have become a key research direction in this field due to their wide sources, excellent biocompatibility, and complete degradability.

In recent years, researchers have carried out a lot of research on polysaccharide-based aerogel filter materials. For example, Yu and Zhang [10] constructed a negatively charged chitosan and phosphorylated cellulose nanofiber aerogel skeleton by freeze-drying, which achieved effective filtration of PM_2.5_ (96.44%) and formaldehyde (23 mg/g). However, they also claimed that the hydrophilic nature of the matrix limited the reusability of the resulting aerogel. Das et al. [11] developed biomass-based graphene aerogels for indoor pollutant removal, achieving outstanding adsorption capacities for HCHO (996.7 mg/g), toluene (392 mg/g), and carbon dioxide (365.3 mg/g), while the missing studies on the mechanical properties might hinder the practical application. Therefore, to develop biomass aerogel materials with high application potential, mechanical strength, filtration efficiency, and service stability should all be taken into consideration, rather than focusing on only the filtration or adsorption function.

Konjac glucomannan (KGM) and sodium alginate (SA), as two typical natural polysaccharides, have attracted considerable attention due to their abundant sources, excellent gel-forming properties, and good biocompatibility [12,13]. KGM molecules contain a large amount of hydroxyl and acetyl groups, which have good water solubility and gelability, but the formed gel structure has poor stability. SA has excellent ionic cross-linking ability and structural stability, and the KGM/SA aerogels could realize synergistic enhancement of properties through hydrogen bonding and electrostatic interaction, making them excellent substrates for constructing green filtration materials [14]. In our previous study, KGM/SA-based polysaccharide aerogels exhibited excellent thermal insulation properties and moderate mechanical strength [13], enabling resistance to airflow impact and alleviation of pore collapse. However, others also reported that KGM/SA aerogels had limited filtration efficiency toward fine particulate matter such as PM_2.5_, and suffered from excessively high pressure drop under high airflow rates [15], restricting their practical application in air filtration. Recently, studies have demonstrated that the incorporation of cellulose fibers can significantly improve the complexity of the pore structure of aerogels as well as mechanical strength [16]. Therefore, the introduction of appropriate functional fibers might regulate the pore structure of KGM/SA aerogels, realizing synergistic optimization of pore structure and air filtration performance.

Silk microfibers (SFs) are biomass protein fibers derived from natural silk with excellent biocompatibility, degradability, and easy functional modification [17,18]. SF molecules contain a large number of active groups, such as amino, hydroxyl, and carboxyl groups, which can not only interact with KGM and SA molecules through hydrogen bonding and electrostatic interactions, but also act as a skeletal support to regulate the pore structure of aerogels by virtue of their natural fibrous structure. Meanwhile, the composite aerogels prepared with SF as the functional component can significantly improve the mechanical strength and structural stability of the materials on the basis of inheriting the advantages of the high porosity and large specific surface area of aerogels [19,20]. Relevant studies showed that SF with a high aspect ratio formed a chimeric or bridging structure with the aerogel matrix [21], significantly improving its mechanical properties and liquid transport efficiency. In addition, Han et al. [22] used silk nanofibers/nitrides to construct porous hierarchical silk nanofiber aerogels with good breathability and mechanical stability. Therefore, the incorporation of SF into a KGM/SA aerogel had high potential to improve the mechanical and air filtration performances.

In this study, aerogels with a cobweb-like porous structure were facilely fabricated by combining natural SF fibers with natural polysaccharides (KGM and SA), which could achieve efficient filtration of multi-scale airborne particulate pollutants. The morphological structures of the aerogels before and after the SF introduction were characterized and analyzed. The effects of SF addition on the pore structure and filtration performance of the aerogels were systematically investigated, and the relationship between pore structure characteristics and air filtration performance was identified. This work provided a theoretical basis and experimental support for the development of eco-friendly and high-performance biomass-based aerogels for air filtration.

## 2. Results and Discussion

### 2.1. Microstructure Characterization of SF

**Figure 1:**
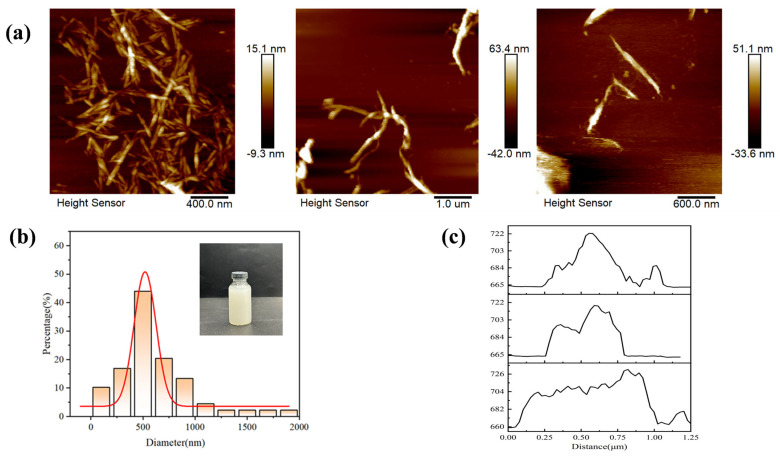
AFM images (**a**), particle size distribution (**b**), and statistics of the size of SF (**c**).

Degummed waste silk fibroin was subjected to swelling treatment in a DES (deep eutectic solvent) solution, followed by mechanical shearing of the swollen silk fibroin. According to the morphology of silk fibroin prior to swelling treatment (Figure S2a in the Supplementary File), obvious sericin and protrusions were observed on the surface of purified silk fibroin, while the surface became smooth and clean after degumming with SF gradually exposed (Figure S2b,c in the Supplementary File). Sericin and impurities on the silk surface were removed by treatment with a sodium carbonate solution, and subsequent swelling treatment was conducted. The DES solution was able to disrupt a portion of the amorphous regions in silk fibroin, break the hydrogen bonds within silk fibroin proteins, and reduce the strength of hydrophobic interactions in the proteins, thus weakening the intermolecular forces between SF fibrils [23]. The remaining fibrils were separated by ultrasonic treatment, yielding SF with a high aspect ratio. Individual fibers were characterized by AFM images (Figure 1a) and particle size measurements (Figure 1b). The DES solution penetrated into the swollen silk fibroin and exfoliated the silk fibers into micro-nano fibers, with the hydrogen bonds between adjacent nano fibers maximally broken [24]. The exfoliated SF exhibited a concentrated diameter distribution centered at 500 nm, which was much smaller than that of natural silk (~13 μm) [25]. The height of individual SF fibers was measured, with an average size of 700 nm (Figure 1c). Due to the dissolution and exfoliation of SF in the DES solvent, SF with a multi-scale hierarchical structure was retained, and incompletely exfoliated structures could occasionally be observed in the AFM images.

### 2.2. Characterization of the Basic Chemical Structure of Silk Microfibers

**Figure 2:**
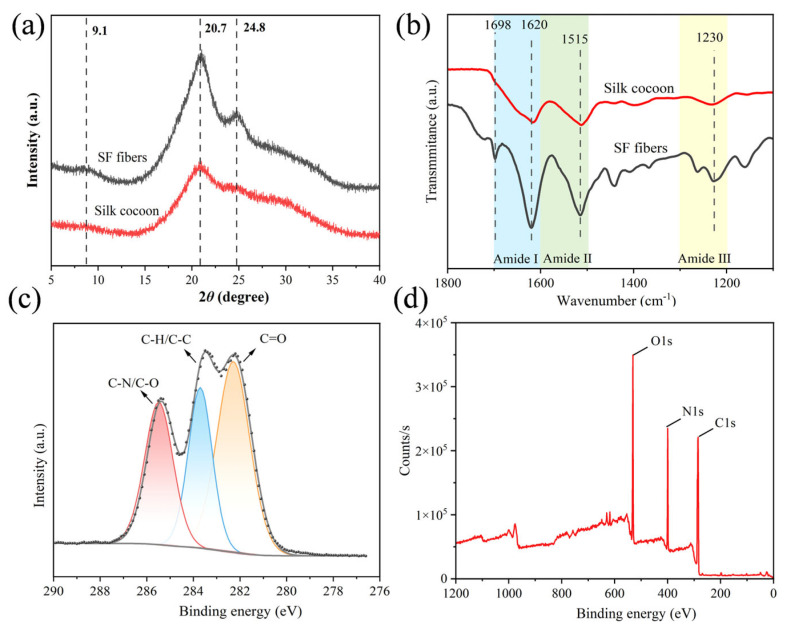
(**a**) XRD pattern of silkworm cocoon and SF; (**b**) FTIR of silkworm cocoon and SF; and (**c**) XPS elemental spectra of silkworm cocoon and SF (peak sections of C1s (**d**) were further analyzed).

To verify that the SF extraction was merely a simple liquid-phase exfoliation without affecting the original structure, the chemical structural stability of SF before and after exfoliation was analyzed and illustrated. The crystalline structures of silk cocoons and SF were characterized by XRD (Figure 2a). The crystalline structure of SF was divided into a Silk I (α-helix) structure and Silk II (β-sheet) structure [26], where the diffraction peaks around 20.7° and 24.8° belonged to the Silk I structure, and the diffraction peak around 9.1° was assigned to the Silk II structure [27]. After swelling and mechanical treatment, no obvious shift of the characteristic peaks of silk fibers was observed, and the intensity of the characteristic peaks increased. This was because the amorphous and non-crystalline regions in the crude silk fibers were removed during the preliminary treatment, resulting in an increase in the crystallinity and peak intensity of SF. In summary, the silk fibers could retain their good crystalline structure after the treatment.

The secondary structure of SF was evaluated by Fourier transform infrared spectroscopy (FTIR) (Figure 2b). In the FTIR spectrum, the characteristic absorption peaks at 1620 cm^−1^, 1515 cm^−1^, and 1230 cm^−1^ were attributed to the β-sheet structure of the amide I, amide II, and amide III bands, respectively. In addition, SF showed a peak at approximately 1698 cm^−1^, corresponding to the β-turn in the amide I band [28]. Compared with the characteristic peaks of SF, no obvious shift of the characteristic absorption peaks of silk cocoons was observed, confirming that both had similar secondary structures. Furthermore, the relative content of the secondary structure in SF fibers was quantitatively analyzed by deconvoluting the FTIR band of the amide I band (1600–1700 cm^−1^) of SF. The relative content of the β-sheet structure was calculated based on the peak area of each sub-peak, and the relative content of the β-sheet structure in SF was 34.83%, which was comparable to that of natural fibers reported in relevant literature (40.47%) [29].

The surface chemical bond state was investigated by XPS scanning (Figure 2c,d), which showed the binding energies of elements O 1s (~530 eV), N 1s (~400 eV), and C 1s (~285 eV). The deconvolution of C 1s could be divided into three peaks, including C=O (282.2 eV), C-H/C-C (284.8 eV), and C-N/C-O (286.7 eV), which is consistent with the XPS C1s deconvolution approach employed by Jain et al. for silk fibroin [30]. The presence of C-C/C-H indicated that there were a large number of non-polar aliphatic carbon skeletons in SF. The peak intensity of C-N was obvious and had a certain ratio to the peak intensity of C=O (usually, the peak area ratio of C-N to C=O in proteins is about 1:1 to 1:1.2), which was a reflection of the characteristic amide bonds (-CONH-) of proteins. The contribution of C-O was small because the content of hydroxyl-containing amino acids (serine, threonine) in SF was not high. By comparison, the proportions of C=O and C-H/C-C were relatively high, which was because SF was in the fiber form and not completely destroyed, and the DES treatment only partially destroyed the amorphous regions on its surface rather than penetrating the interior of the fibers [21]. This would improve the relative roughness, crystallinity, and β-sheet content of SF, while more C-O and C=O groups were exposed on its surface.

### 2.3. Microstructure Characterization of KSSF Composite Aerogels

**Figure 3:**
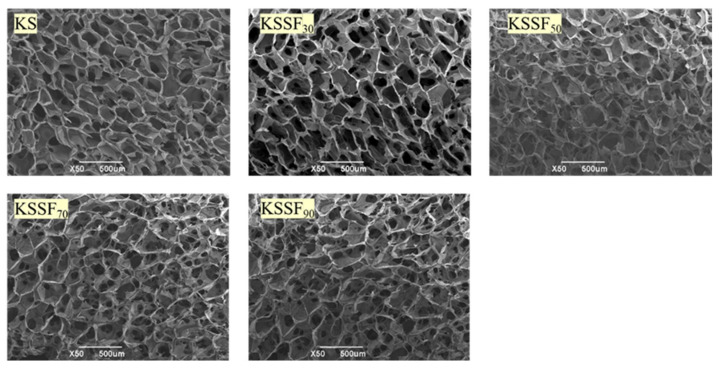
SEM images of KS and KSSF composite aerogels.

The microstructure of KSSF composite aerogels with different SF addition amounts was characterized (Figure 3), and the pore size was analyzed preliminarily using ImageJ software (version 1.8.0.345; Figure S3 in the Supplementary File). All samples exhibited a three-dimensional porous structure typical of aerogels, with KSSF aerogel pore sizes decreasing and concentrated in the range of 50–100 μm. Compared with the smooth pore walls of neat KS aerogels, the KSSF aerogels with SF content from 30% to 70% showed uniformly distributed pores without obvious macropore defects or structural collapse, maintaining an intact polysaccharide skeleton. This phenomenon can be attributed to the formation of aerogel pores via ice crystal growth and subsequent sublimation during freeze-drying. In the pure KGM/SA system, the mechanical strength of the polysaccharide crosslinked network was limited, making ice crystals prone to growing into large crystals during freezing and thereby forming large, inhomogeneous pores. After introducing SF, the uniformly dispersed SF effectively restricted the growth size of ice crystals, leading to pore size refinement. Meanwhile, the amino and hydroxyl groups in SF molecules formed intermolecular hydrogen bonds with the hydroxyl and carboxyl groups of KGM/SA, which enhanced the collapse resistance of the gel skeleton and prevented pore structural deformation during drying, ensuring pore uniformity. These positive impacts appeared to be the strongest at the SF addition of 70%. However, when the SF content further increased to 90%, the pore structure of the aerogel was damaged, and the pore morphology became disordered. This was likely due to spontaneous aggregation of excessive SF fibers, which disrupted the intrinsic three-dimensional network structure of the KGM/SA polysaccharide matrix.

**Figure 4:**
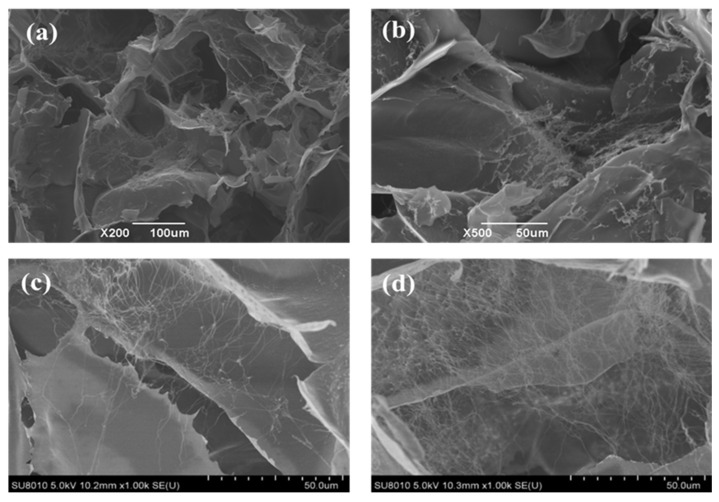
Local magnified SEM images of KSSF_70_ composite aerogels with different magnifications (×200 (**a**), ×500 (**b**), and ×1000 (**c**,**d**)).

The local pore structure of the KSSF_70_ aerogel was further observed under high-magnification SEM (Figure 4a,b). It was found that SF mostly adhered to the pore walls and extended into the cavities, showing a spider-web-like distribution in the pores (Figure 4c,d). A three-dimensional aerogel framework structure was constructed by freeze-induced and vacuum freeze-drying methods. During the freezing process, the SF in the solution was squeezed away by the temperature-induced moving water-ice crystals and accumulated in the gap regions of the growing ice crystals (Figure 4a,b) [28]. According to related studies, the ice crystals formed in frozen polysaccharide sols were approximately 50 μm in size (e.g., locust bean gum [31], KGM/starch blends [32]). However, the SF surface treated with DES exposed a large number of amino acid residues, making the formation of intermolecular hydrogen bonds within SF easier, and SF was directly entangled with itself. Meanwhile, the hydrophilic groups in SF absorbed a small amount of water molecules, forming tiny ice crystals during freezing, and after the ice sublimation, SF was fixed in the pore walls and pores of the aerogel [33]. Therefore, the three-dimensional pore walls of the aerogel were constructed through the entanglement of interconnected SF and the hydrogen bonds formed between SF and polysaccharide molecules (KGM/SA). Similarly, Yang et al. [34] proposed that the distribution characteristics of SF were most likely attributed to the distinct motion behaviors of SF when interacting with growing ice crystals. SF molecules can be pushed, bypassed, and anchored by continuously growing ice crystals, thereby becoming immobilized on the polysaccharide pore walls and forming the spider-web-like structure observed.

### 2.4. FTIR Spectra

**Figure 5:**
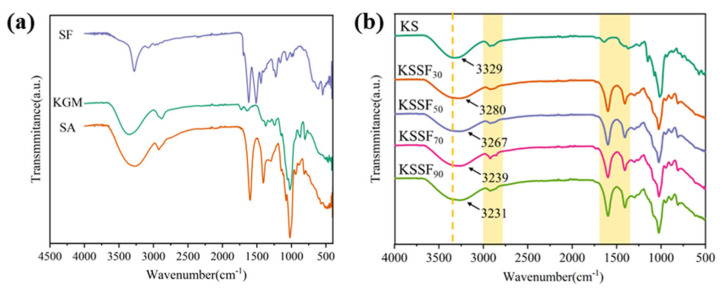
Fourier transform infrared spectra of component materials (**a**) and KSSF aerogels (**b**).

The FTIR spectra of both component materials and KSSF aerogels were analyzed (Figure 5). Specifically, SF had typical Silk II structure characteristic peaks at 1620 cm^−1^ (amide I, β-sheet structure) and 1515 cm^−1^ (amide II, β-sheet structure), and the stretching vibrations of methyl (-CH_3_) and methylene (-CH_2_-) groups were between 2800 and 3300 cm^−1^ [35]. The main characteristic absorption peak in KGM was located at 1022 cm^−1^, corresponding to the stretching vibration of the sugar ring C-O-C (β-1,4-glycosidic bond). The 3000–3500 cm^−1^ range corresponded to the O-H stretching vibrations within or between polysaccharide molecules. Because KGM contained a large number of hydroxyl groups, the intensity and width of this peak were significantly higher than those of SF [36]. SA molecules contained a large number of sodium carboxyl groups (-COO^−^), and the double peak at 1400–1650 cm^−1^ corresponded to the symmetric and asymmetric stretching vibrations of carboxylate -COO^-^.

As KGM and SA had abundant hydroxyl groups in their molecular structures, a stable framework structure was mainly formed through hydrogen bond interactions, which related to stretching vibration absorption peaks (3300–3500 cm^−1^) of hydroxyl groups (-OH). Wang et al. [37] analyzed the FTIR spectra of SF/SA composite films, which also confirmed the existence of intermolecular hydrogen bonds between these two components. The peaks gradually shifted to the lower wavenumbers with greater SF addition, indicating that the hydrogen bond interactions between SF and composite polysaccharides were enhanced. Similarly, Poluri et al. [38] observed a shift of the characteristic absorption bands in the FTIR spectra of a zein/chitosan protein–polysaccharide composite system, which was attributed to the formation of intermolecular hydrogen bonds between the two components. On the other hand, compared with KS aerogels, the peak intensity of KSSF aerogels in the range of 1300–1600 cm^−1^ showed a stronger trend. This might be the asymmetric/symmetric stretching vibrations of the carboxyl groups of SA being overlapped with the amide II band of SF, leading to the enhanced peak at this position, indicating a stable interaction in the SF/KGM/SA composite system.

### 2.5. XRD and XPS Characterization of KSSF Composite Aerogels

**Figure 6:**
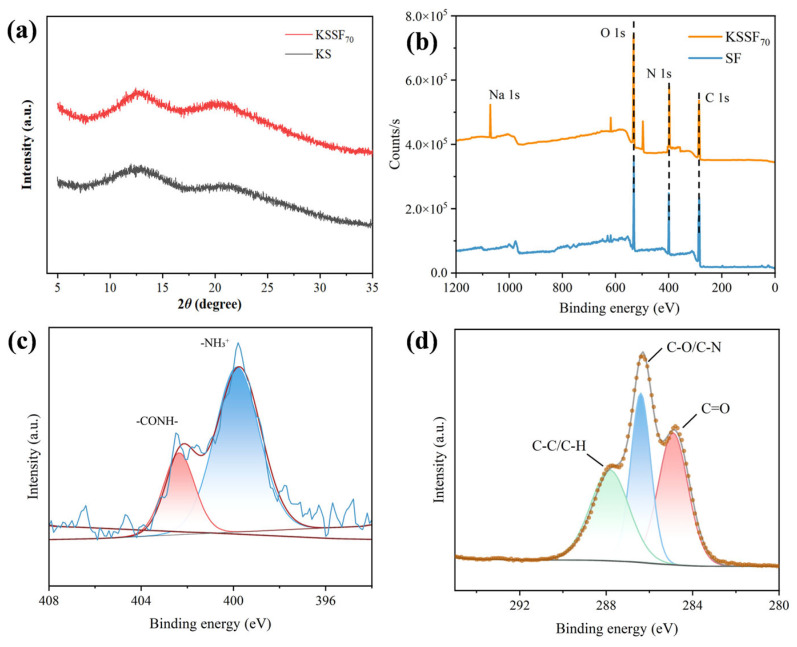
(**a**) XRD of KS and KSSF_70_ aerogels; (**b**) XPS elemental spectra of KSSF_70_ aerogel and SF (peak sections of N 1s (**c**) and C 1s (**d**) were further analyzed).

The basic chemical structures of pure polysaccharide KS aerogels and KSSF composite aerogels were characterized by XRD and XPS. According to the XRD diffraction pattern (Figure 6a), KS and KSSF_70_ aerogels did not have sharp crystalline diffraction peaks, and only exhibited broad diffuse diffraction halos at around 2θ = 12° and 20°, indicating that both samples possessed a dominant amorphous structure, which belonged to a typical structural feature of natural polysaccharide-based aerogels [39]. After the SF incorporation, the overall diffraction intensity of KSSF_70_ was increased with a more distinct peak shape. However, the addition of a small amount of SF did not alter the amorphous nature of the aerogel, which was conducive to the formation of a continuous and stable three-dimensional interpenetrating network structure [40].

The full XPS spectra (Figure 6b) were further analyzed to investigate the changes in surface elemental composition and chemical state of the aerogels. Both spectra showed a C 1s peak at 285 eV, N 1s peak at 400 eV, and O 1s peak at 532 eV. Compared with SF, the KSSF aerogel had a weak Na 1s signal at 1072 eV, which was due to the presence of sodium ions in SA [41]. Meanwhile, the intensity of the N 1s peak in KSSF decreased, while the intensity of the O 1s peak relatively increased. This was because the major components of KSSF were KGM and SA, both of which were polysaccharides containing a large number of hydroxyl groups (-OH) and carboxylate groups (-COO^−^) but without nitrogen, resulting in a decrease in the proportion of surface N elements and a high O content.

Deconvolution of N 1s and C 1s spectra (Figure 6c,d) was additionally performed to clarify the chemical bonding states and functional groups of the aerogel. Results showed that the N 1s XPS peak of KSSF_70_ deconvoluted into two characteristic peaks, which were assigned to an amide bond (-CONH-, ~402.0 eV) and protonated amino group (-NH_3_^+^, ~400.0 eV). As KGM and SA did not contain nitrogen, the N 1s signal came entirely from SF, confirming the successful introduction of SF in the composite aerogel. The shape of the C 1s peak changed, and deconvolution treatment was performed on it (Figure 6d). C-C/C-H (284.8 eV) mainly came from aliphatic chains, aromatic rings or carbon skeletons in proteins; C-O (286.5 eV) came from hydroxyl groups and ether bonds of KGM and SA, and C-N came from amide bonds or amino groups of SF, agreeing with Nashed et al. [42]; and C=O, a typical peak of high oxidation state carbon, came from amide bonds of proteins, ester bonds of polysaccharides or sugar ring structures [43]. Compared with SF (Figure 2c), the C-O/C-N peak dominated in the C 1s spectrum, and C=O mainly came from amides. After compounding with KGM/SA, both the C-O/C-N and C=O peaks in the C 1s spectrum of KSSF were enhanced, indicating that the surface of KSSF had both oxygen-containing functional groups of polysaccharides and amide functional groups of proteins. This demonstrated the homogeneous mixing of the two components. Meanwhile, the coexistence of characteristic functional groups demonstrated good interfacial compatibility between SF and the polysaccharide matrix in the as-prepared aerogel network [44].

### 2.6. Mechanical Properties of KSSF Composite Aerogel

**Figure 7:**
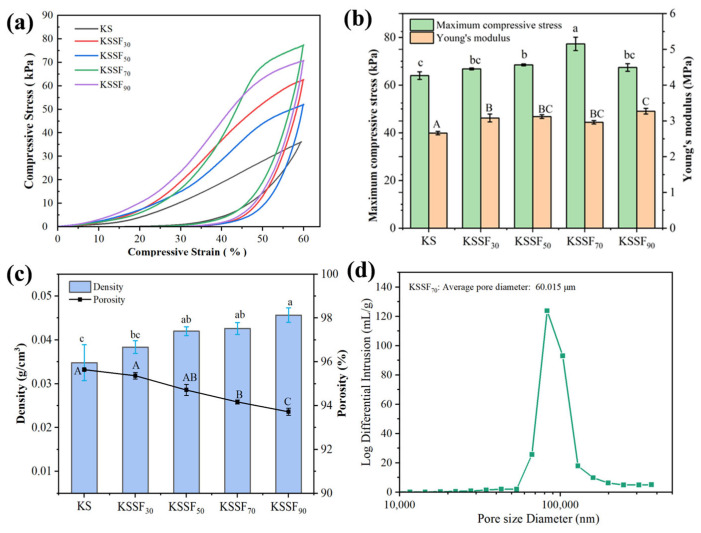
(**a**,**b**) Mechanical properties of KS and KSSF aerogels ((**a**) compressive stress–strain curve; (**b**) maximum compressive stress and Young’s modulus); (**c**) density and porosity of KS and KSSF aerogels; and (**d**) pore size distribution of KSSF_70_ aerogel. Data marked with the same letters indicate no significant differences at *p* < 0.05.

The mechanical properties of the KSSF composite aerogels were characterized via compression tests (Figure 7a,b), indicating that the maximum compressive stress and Young’s modulus of all KSSF aerogels increased with higher SF addition. Figure 7c revealed that the porosity of all samples remained above 93%, while the density of KSSF aerogels increased with higher SF contents. This could partially explain the gradually stronger maximum compressive stress of KSSF aerogels with SF increased from 0% to 70%. At this SF addition range, the polysaccharides were entangled and linked with SF to form fibrous pore wall supports, and simultaneously constructed a continuous and intact interpenetrating network (i.e., a cobweb-like pore structure) inside the pores. Upon stress application, the polysaccharide pore walls and the attached SF jointly bore the stress, accompanied by slight deformation of the pore structure. This effectively avoided overloading the polysaccharide network, thereby improving the maximum compressive stress [45,46]. However, a negative impact on maximum compressive stress was observed when the SF addition was increased to 90%. According to the previous SEM images (Figure 3), the pore structure of KSSF_90_ was destroyed due to the excessive aggregation of SF fibers, leading to pore wall rupture and deformation of the pore structure and the formation of stress concentration points, which ultimately resulted in the reduction of compressive strength. Therefore, the highest maximum compressive stress (78.89 kPa) was achieved for KSSF_70_.

To further illustrate the mechanical property, the Young’s modulus was also calculated. Within the linear elastic region (<15%), with increasing SF content, the Young’s modulus of the KSSF aerogels was increased, leading to enhanced deformation resistance. Above 20% strain, the stress–strain curves of all KSSF aerogels show a steeper slope and a faster stress increase compared with the pure KS aerogel. This was because the SF-reinforced network enhanced skeleton rigidity and suppressed pore collapse, enabling more efficient structural densification and improved load-bearing capacity under compression. Notably, KSSF_70_ showed a relatively low Young’s modulus (Figure 7b), which could be attributed to the amorphous structure (random coils in amorphous regions) and α-helices of SF attached to the pore walls. These structures provided more recoverable elastic supporting points, preventing pore collapse during compression and imparting certain resilience to the aerogel, which was found to have a certain buffering effect [47]. Therefore, with further higher strain (≥40%), the KSSF_70_ sample could still maintain a relatively intact pore wall structure against the compression.

The pore size distribution of the KSSF_70_ aerogel obtained from mercury intrusion porosimetry is shown in Figure 7d. It had an average pore size of 60.015 μm, and the curve displays a unimodal distribution without distinct separated peaks for multi-scale pores, suggesting a concentrated pore size distribution and a prominent proportion of pore volume in the aerogel. The uniform pore size allowed stress to be transferred uniformly throughout the entire skeleton, preventing local stress concentration. The fractal dimension was determined to be 2.999 (Figure S4 in the Supplementary File), demonstrating that the pore structure of the KSSF aerogel possessed highly complex fractal features with a high degree of pore space filling, which was beneficial to its performance in air filtration, pollutant adsorption, and other related applications.

### 2.7. Filtration Performance of KSSF_X_ Composite Aerogels

**Figure 8:**
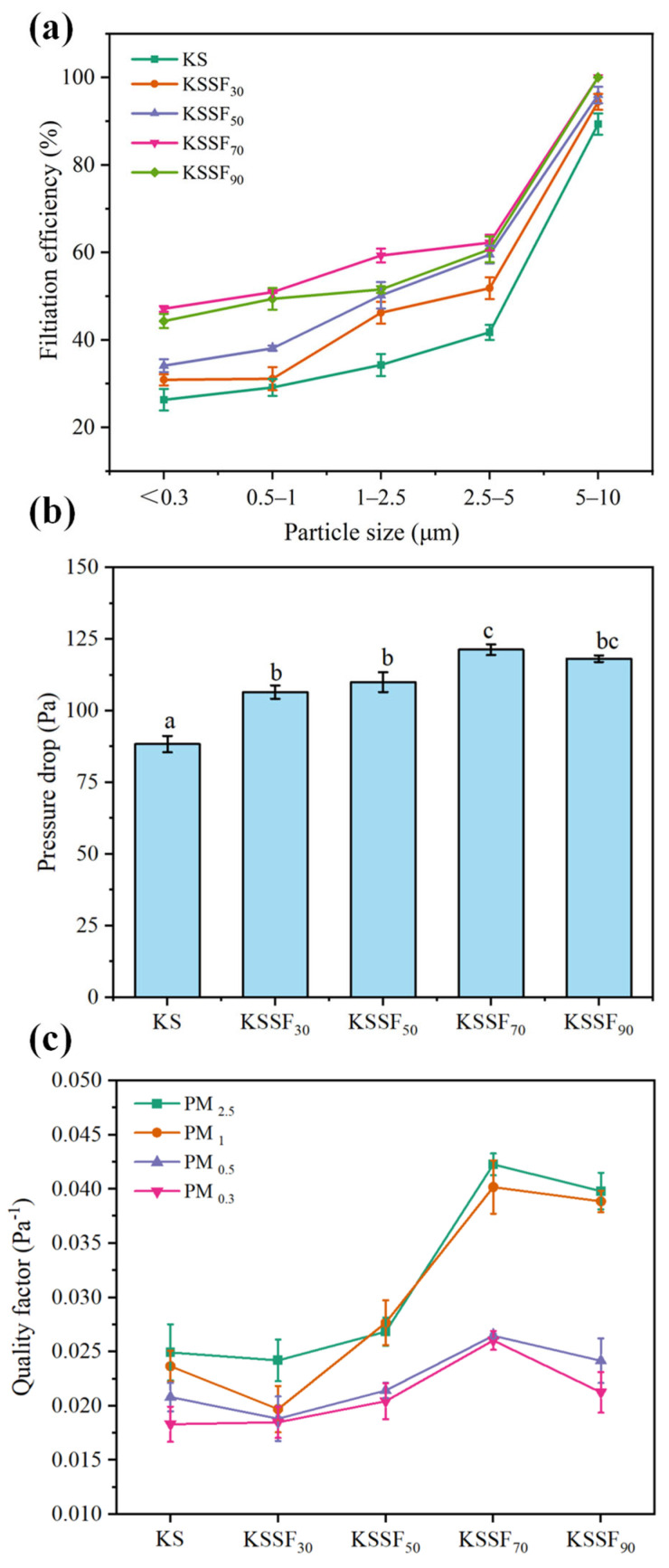
Filtration efficiency (**a**), pressure drop (**b**), and QF (**c**) of KS and KSSF composite aerogels. Data marked with the same letters indicate no significant differences at *p* < 0.05.

The air filtration performance of the composite aerogels was evaluated using a comprehensive filter test bench, with the filtration efficiency and pressure drop toward particulate matters of different sizes recorded (Figure 8a,b). With increasing SF addition, the filtration efficiency of KSSF aerogels for particulate matter across various size ranges exhibited an overall trend of rising first and then declining. When the SF addition was increased from 0 to 70%, the filtration efficiency in each particle size range, especially for fine particulate matter such as <0.3 μm and 0.5~1 μm, improved continuously and markedly. The filtration efficiency of KSSF_70_ for particles < 0.3 μm increased from 26% in the KS group to 47%, its removal efficiency for PM_2.5_ reached 71.2%, and the filtration efficiency for large particles of 5~10 μm was close to 100%. When the SF addition was further raised to 90%, the filtration efficiency for fine particulate matter (<2.5 μm) became slightly lower than that of KSSF_70_, with only a minor increase observed in the efficiency for large-sized particles. According to Figure 8b, the airflow pressure drop of the aerogels first increased and then decreased with increasing SF addition. The pressure drop of KSSF_70_ reached the peak value (122 Pa) among all samples, while that of KSSF_90_ was slightly lower than that of KSSF_70_. This was attributed to the gradually reduced porosity of the aerogels caused by the introduction of SF. Meanwhile, SF fibers formed a three-dimensionally intertwined cobweb-like structure within the pore channels, which enhanced the capture capability for particulate matter through multiple mechanisms, including interception, impaction, and diffusion-sieving [48]. Meanwhile, the denser pore structure increases airflow resistance, leading to a higher pressure drop. When the SF addition reached 90%, fiber agglomeration occurred, resulting in deteriorated pore uniformity and an increase in local macropores. This weakened the interception effect toward fine particulate matter, while airflow passed more readily through macroporous channels, causing a slight decline in pressure drop.

To comprehensively evaluate the filtration performance of aerogels, the KSSF aerogel mass factors of PM_0.3_, PM_0.5_, PM_1_, and PM_2.5_ were calculated (Figure 8c) [49]. For PM_5-10_ coarse particles with filtration efficiency close to 100%, the reference value of the mass factor in performance evaluation is limited, so we focused on early fine particulate matter (PM_0.3_–PM_2.5_). The results demonstrated that KSSF_70_ exhibited the highest QF for particulate matter of all tested sizes among all samples (the QF value of KSSF_70_ for PM_2.5_ was 0.042), indicating that it achieved the optimal balance between high filtration efficiency and low pressure drop, making it the sample with the best comprehensive filtration performance in this system. Further comparisons of QF for the coarse particles (PM_5–10_) also showed the same change trend (Figure S5 in the Supplementary File), and therefore, KSSF_70_ had the best performance across all particle sizes. This can be attributed to the uniform three-dimensional porous structure and continuous fiber-reinforced network of KSSF_70_, which not only enabled efficient interception of particulate matter through refined pore sizes and interwoven fibers but also avoided pore channel blockage and a sharp rise in airflow resistance caused by excessive fiber agglomeration, thus balancing filtration efficiency and air permeability [50]. Compared with the KGM/gelatin aerogel reported by Wang et al. [51], which also utilized eco-friendly macromolecules and achieved a PM_10_ filtration efficiency of approximately 92%, the KSSF_70_ aerogel in this study delivered a significantly enhanced PM_10_ filtration efficiency of 99.8%. Furthermore, while sharing a comparable PM_10_ filtration efficiency with the pure silk fibroin aerogel from Yang et al. [52], the KSSF_70_ aerogel provided a higher compressive stress at 60% strain.

## 3. Conclusions

In this study, a series of KSSF composite aerogels was successfully fabricated via freeze-drying, with silk microfibers (SFs) as reinforcement and konjac glucomannan/sodium alginate (KGM/SA) polysaccharides as the matrix. SF showed a concentrated diameter of 500 nm and retained the crystalline and β-sheet secondary structures of natural silk. SF addition effectively regulated the pore architecture of KGM/SA aerogels. At low to medium loadings (30%~70%), the aerogels exhibited reduced pore size and more regular morphology. Among all samples, KSSF_70_ had the optimal mechanical properties (maximum compressive stress: 78.89 kPa) and a regular cobweb-like pore structure, which enabled efficient particle interception and maintained structural stability against particle deposition and high-velocity airflow during filtration. Correspondingly, KSSF_70_ achieved 99.8% filtration efficiency for PM_10_ and 71.2% for PM_2.5_, and a QF that complied with mainstream commercial filters. This work provided a feasible strategy for developing biodegradable polysaccharide-based composite aerogels with comprehensively enhanced mechanical strength and air filtration performance with SF addition, showing promising potential in green air purification applications. Further investigations on the stability and durability of the filtration performance over multiple filtration cycles can be conducted to verify its practical performance, and the production process should also be optimized to reduce the cost, considering market competitiveness. Meanwhile, introducing antimicrobial activity might also be considered to further improve the air quality.

## 4. Materials and Methods

### 4.1. Experimental Materials

KGM (purity ≥ 95%; Mw = 1.01 × 10^6^ g/mol; molar ratio of mannose:glucose of 1.6:1; acetylation degree 1.85%) was purchased from Hubei Konson Konjac Technology Co., Ltd. (Wuhan, China). SA (analytical grade, Mw = 2.2 × 10^5^ g/mol) was purchased from Macklin Biochemical Co., Ltd. (Shanghai, China). Sodium carbonate, malonic acid (MA), and choline chloride (ChCl) were all analytical grade and were purchased from Macklin Biochemical Co., Ltd. (Shanghai, China). SF was purchased from Hualide Technology Co., Ltd. (Beijing, China).

### 4.2. Extraction and Preparation of SF Microfibers

The SF extraction method from Tan et al. [53] was employed with proper modifications. The dissolution of silk fibroin uses protein denaturants to break the strong intermolecular hydrogen bonds within silk fibroin, enabling the conversion of silk fibroin fibers into an aqueous silk fibroin solution or a silk fibroin nanofiber suspension [54]. Deep eutectic solvent (DES), a class of green and customizable eutectic mixtures formed by hydrogen bond acceptors (HBAs) and hydrogen bond donors (HBDs), was used to exfoliate degummed silk cocoons [55]. The hydrogen bonding system of DES can effectively compete with the intrinsic intermolecular hydrogen bonds of silk fibrin, form new hydrogen bonds with the amino, hydroxyl, and carbonyl groups of silk fibrin proteins, break the original hydrogen bonds of the protein itself, and dismantle the dense aggregate structure of silk, to gently dissolve and peel off silk fibers without damaging the secondary structure of proteins. Specifically, the DES used in this work was prepared by mixing choline chloride (HBA) and malic acid (HBD) at a molar ratio of 1:1, which was heated at 90 °C under stirring to form a transparent and homogeneous solution.

Empty silk cocoons were cut into pieces and added to a boiling 0.5 wt% sodium carbonate aqueous solution for degumming for 30 min, followed by washing with deionized water to remove residual sericin, before being dried at 60 °C for 12 h. Dried ChCl and MA were mixed at a molar ratio of 1:1 and heated at 90 °C to obtain a clear DES solution. ChCl and MA were able to break the non-covalent bonds (e.g., hydrogen bonds) in biomass materials and reduce the strength of hydrophobic interactions in proteins. The crude silk fibers obtained from degumming were added to the DES solution at a weight ratio of 1:100, and the mixture was vigorously stirred at 100 °C for 36 h to form a paste-like mixture. Anhydrous ethanol was added at a volume ratio of 1:2 (ethanol: DES) to disperse the DES into anhydrous ethanol, and the resultant mixture was centrifuged at 8000 rpm for 10 min to separate the DES-treated silk fibroin from the DES/ethanol mixed solution. The separated DES-treated silk fibroin was added to deionized water at a weight ratio of 1:100 and treated with an ultrasonic disruptor for 30 min to prepare a 1 wt% SF suspension.

### 4.3. Preparation of KGM/SA/SF Aerogels

According to Wu et al. [13], with slight modifications, a certain amount of SF suspension was first dispersed in 100 g of deionized water, followed by the addition of KGM (0.75 g) and SA (1.50 g). The mixture was heated to 60 °C with a constant stirring speed of 600 rpm for 1 h to obtain a mixed sol. The obtained sol was injected into a six-well culture plate (φ = 4.0 cm; h = 1.5 cm), placed in a constant temperature refrigerator at 4 °C for pre-cooling, and the temperature was monitored using a T-type thermocouple. When the temperature reached 0 °C, the plate was taken out and immediately placed in an ultra-low temperature refrigerator at −20 °C for 12 h. Subsequently, the frozen sample was freeze-dried in a vacuum freeze-dryer with a cold trap temperature of −50 °C and a vacuum degree of 10 Pa for 48 h to obtain the composite aerogel. For the air filtration test, the aerogel samples (thickness of approximately 3 mm) were obtained using a circular culture dish (diameter φ = 15.0 cm; h = 1.5 cm) instead of the six-well plate to meet the size requirements (Figure S1 in the Supplementary File). The obtained samples were coded as KSSFx (x indicated the SF contents). For example, KSSF_70_ indicates that the SF content is 70% (*w*/*v*) based on the solid content.

### 4.4. Microstructure Observation

#### 4.4.1. High-Resolution Scanning Electron Microscopy (SEM)

The diluted SF suspension (0.01 wt%) was dropped onto the surface of a clean silicon wafer and air-dried. Then, the wafer was cut into 5 mm × 5 mm pieces and was affixed to the sample stage with conductive adhesive. After gold sputtering, the micro-morphologies of the samples were observed via SEM (JSM 6390LV, JEOL, Tokyo, Japan) at an accelerating voltage of 15 kV. The cross-sections of the cut KSSF samples (5 mm × 5 mm × 1 mm) were affixed to the sample stage with conductive adhesive, before being observed via the same SEM after gold sputtering.

#### 4.4.2. Atomic Force Microscopy (AFM)

A Bruker ICON6-SYS atomic force microscope (Dimension Icon, Bruker, Berlin, Germany) equipped with a diamond-coated tapping mode probe (DT-NCHR, Nanosensors, Bruker, Berlin, Germany) was employed. The diluted SF fiber suspension (15 μL) was dripped onto mica sheets using a pipette and dried at 25 °C for 12 h. Then, it was observed with a scanning area of 5 μm × 5 μm, a scanning frequency of 0.800 Hz, and a scanning pixel resolution of 256 × 256. NanoScope Analysis 1.8 software was used to analyze the images.

### 4.5. Particle Size Testing

Diluted SF dispersion (1 mg/mL) was prepared and loaded into the laser particle size analyzer (ZEN3600, Malvern, UK) to obtain the particle size of the SF.

### 4.6. X-Ray Diffraction (XRD) Measurements

A Bruker X-ray diffractometer (Karlsruhe, Baden-Württemberg, Germany) was employed to characterize the SF and KSSF aerogels. The test parameters were set as follows: Cu Kα target with a characteristic wavelength of 0.154 nm, an operating voltage of 45 kV, and an operating current of 40 mA, a diffraction angle (2θ) scanning range of 5~40°, a scanning step size of 0.02°/s, and a scanning speed of 2°/min.

### 4.7. Attenuated Total Reflection Fourier Transform Infrared Spectroscopy (ATR-FTIR)

The functional group differences of SF and KSSF aerogels were investigated by ATR-FTIR on a Nicolet iS 10 spectrometer (Thermo Fisher Scientific, Madison, WI, USA). Before the test, the samples were dried in an oven at 60 °C for 12 h to remove moisture interference. The scanning wavelength range was 500~4000 cm^−1^ with a resolution of 4 cm^−1^, and each sample was scanned 3 times (number of runs = 3). Data were processed using OMNIC 8.2 software (Thermo Fisher Scientific) with baseline correction and Savitzky–Golay smoothing (window size = 11 points) to eliminate noise.

### 4.8. X-Ray Photoelectron Spectroscopy (XPS) Test

An ultra-high vacuum X-ray photoelectron spectrometer (Thermo Scientific K-Alpha, Thermo Fisher Scientific, Madison, WI, USA) was employed to analyze the chemical elemental compositions of the SF and KSSF aerogels. The test parameters were set as follows: the take-off angle of photoelectron emission was 90°, a monochromatic Al Kα target with an excitation energy of 1486.68 eV was used, and the charging effect was corrected by taking the C 1s peak of hydrocarbons at 284.8 eV as the reference. For data processing, peak fitting was conducted via the Avantage 6.9.0 software (Thermo Fisher Scientific).

### 4.9. Density and Porosity

The mass (*m*) of KSSFx aerogels was measured using an analytical balance (ME204, METTLER TOLEDO, Changzhou, China). The volume (*V*) of the aerogels was calculated from their diameter and height. The bulk density (ρ) of the aerogels was further calculated according to the following equation (Equation (1)):

(1) $$\rho =\frac{m}{v}$$

The porosity of the aerogels was determined based on the method reported in the previous research of our laboratory [56]. An aerogel sample was weighed (denoted as *m*_0_) and immersed in a container filled with ethanol, with the total weight of the container and its contents recorded as m_1_. The container was then subjected to vacuum treatment until no air bubbles escaped from the ethanol. After the sample was taken out of the container, the weight of the container with residual ethanol was recorded as *m*_2_. The porosity of the sample was subsequently calculated according to the following equation (Equation (2)):

(2) $$porosity\left(\%\right)=\frac{m1-m2-m0}{m1-m2}\times 100$$

In addition, a mercury intrusion porosimeter (AutoPore IV 9520, Micromeritics, Norcross, GA, USA) was employed to perform mercury intrusion tests on the KSSF aerogels. The test parameters were set as follows: a mercury intrusion pressure range of 0.10~61,000.0 psia and a mercury intrusion temperature of 24.00 °C. The pore structure and physical properties of the aerogels were characterized through this test.

### 4.10. Mechanical Properties

Compression tests were carried out on a texture analyzer (TA.XT Plus, Stable Micro Systems, Surrey, UK) equipped with a 30 kg load cell and a discoidal probe (P/36R, No. 14631), based on a previous method [16], modified as follows. Specifically, the trigger force was set to 1.0 N, and the compression rate and compression percentage were 1.00 mm/s and 60%, respectively. The maximum compressive stress (σ; kPa) of the specimen was calculated by (Equation (3)). The Young’s modulus (MPa) was obtained by fitting the slope of the curve in the linear elastic region (strain 0~15%) of the stress–strain curve using texture analysis software.

(3) $$\sigma =\frac{F}{S_{0}}$$

where *F* (N) is the maximum compressive force during the test, and *S*_0_ (mm^2^) is the test area of the sample.

### 4.11. Filtration Performance Testing

Filtration tests were performed using methods similar to those reported in our previous work under ambient conditions [32] (temperature = 25 ± 2 °C, relative humidity = 50 ± 5% RH). The filtration efficiency (*η*) and filtration resistance (shown as pressure drop, Δ*P*) of the aerogel samples were characterized using an automated filter tester (LZC-K1, Suzhou Huada Instrument Co., Ltd., Suzhou, China). The particles were generated by an aerosol generator (SLG 250, Topas, Dresden, Germany) using neutral monodisperse NaCl aerosol particles (0.26 μm in diameter). Tests were performed with a gas flow rate of 32 L/min calibrated using an air velocity meter (Airflow Instruments Velocimeter TA430, TSI Instruments Ltd, Shoreview, MN, USA). The effective test area of all samples was 15 cm in diameter. Each sample was tested 3 times. The filtration efficiency was determined as (Equation (4)):

(4) $$\eta \left(\%\right)=\frac{C_{2}-C_{1}}{C_{2}}\times 100$$

where *C*_1_ and *C*_2_ are the number of particles before and after filtration, respectively. Δ*P* is the difference in pressure drop between upstream and downstream pressures.

In order to evaluate the comprehensive filtration performance of the filter material, QF was used to normalize the pressure drop and filtration efficiency as follows [57] (Equation (5)):

(5) $$QF=\frac{-ln\left(1-\eta \right)}{\Delta P}$$

### 4.12. Statistical Analysis

All tests were performed at least in triplicate. Figures were drawn using Origin 2021 Pro (Origin Lab Corporation, Northampton, MA, USA). Data were subjected to a one-way ANOVA test by Tukey’s analysis using IBM SPSS Statistics 25 (Chicago, IL, USA), and the significance level was at *p* < 0.05. Different letters represent significant differences between samples.

## Data Availability

Data will be made available on reasonable request.

## Supplementary Materials

The following supporting information can be downloaded at https://www.mdpi.com/article/10.3390/gels12050443/s1: Figure S1: Preparation process of KSSFx composite aerogel; Figure S2: SEM morphology of silk before (a) and after degumming (b), polarized microscopic image of silk fiber in aqueous solution (c), SEM image of SF after DES treatment (d); Figure S3: Pore size distribution of KSSF aerogels with different SF additions analyzed by ImageJ (for each sample, three representative SEM images (×50) were used for the pore size analysis); Figure S4: Pore size fractal dimension of KSSF_70_ aerogel obtained from mercury intrusion testing (the theoretical range of the fractal dimension is 2–3, where two corresponds to an ideally smooth planar structure and three corresponds to an extremely complex structure that completely fills space). Figure S5: Mass Factor (QF) of KS and KSSF Aerogels of PM_5_ and PM_10_.

## Abbreviations

The following abbreviations are used in this manuscript:

KGM	Konjac glucomannan
SA	Sodium alginate
SF	Silk microfiber
KSSF	KGM/SA/SF aerogel
KSSF_x_	Sample codes of KSSF aerogels (x indicates the SF contents)
