# Supplementary material for: Silk Microfiber-Reinforced Biomass Aerogel with Cobweb-like Pore Structure for Highly Efficient Eco-Friendly Air Filtration
Source: Gels. 2026 May 19;12(5):443. doi: 10.3390/gels12050443 (PMC13205734; doi:10.3390/gels12050443)
Supplement: Supplementary file 1 [file gels-12-00443-s001.zip › gels-4300994-supplementary.pdf]

Supplementary materials for "Silk microfiber-reinforced biomass aerogel with cobweb-like pore structure for highly efficient eco-friendly air filtration"

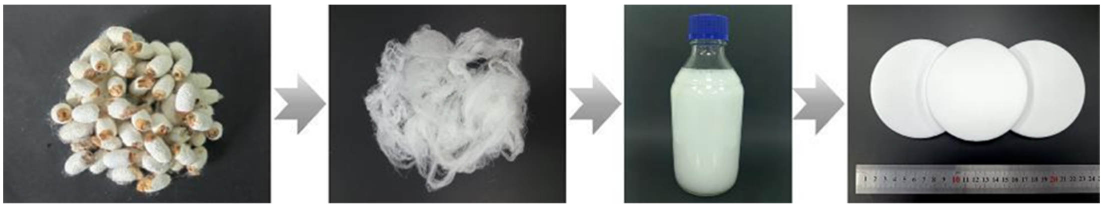

Figure S1 Preparation process of $KSSF_x$ composite aerogel

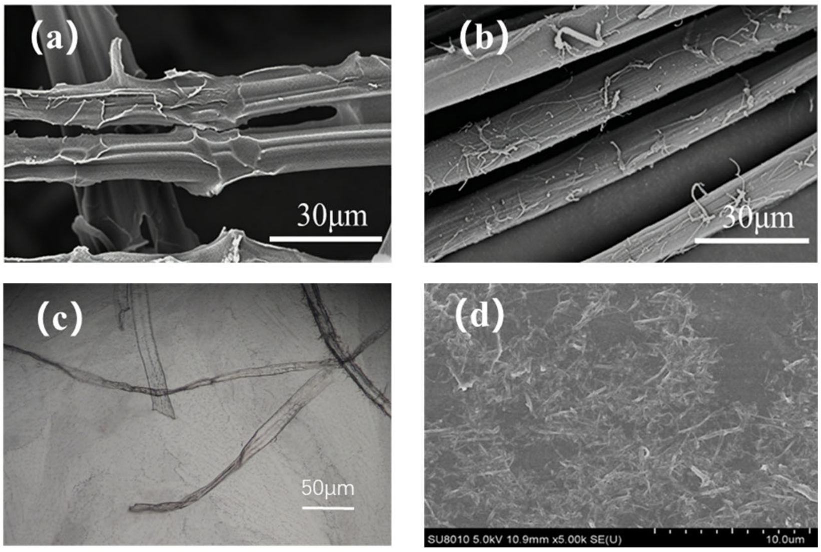

Figure S2 SEM morphology of silk before (a) and after degumming (b), polarized microscopic image of silk fiber in aqueous solution (c), SEM image of SF after DES treatment (d)

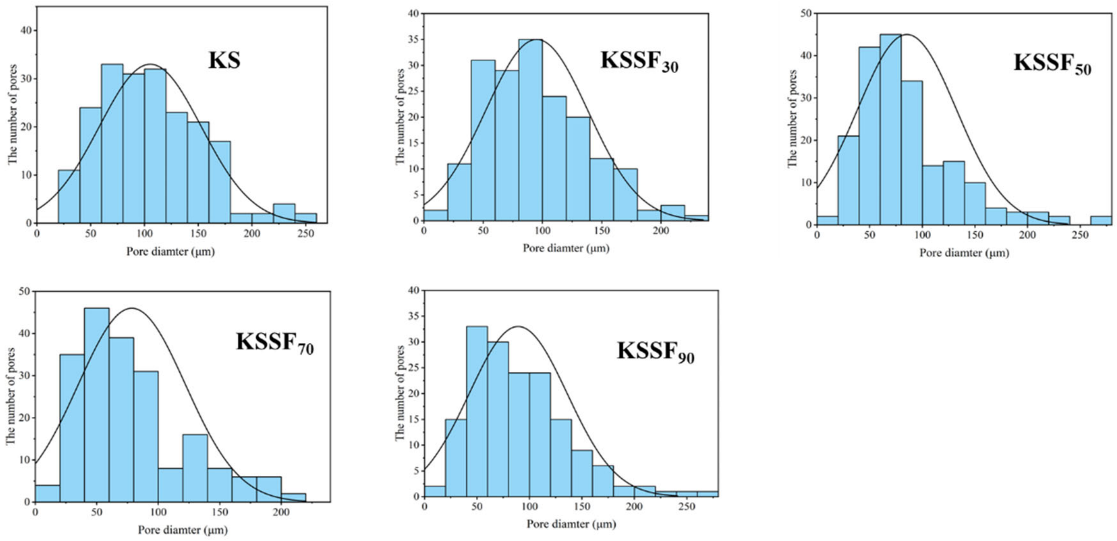

Figure S3 Pore size distribution of KSSF aerogels with different SF additions analyzed by ImageJ software (For each sample, three representative SEM images (×50) were used for the pore size analysis)

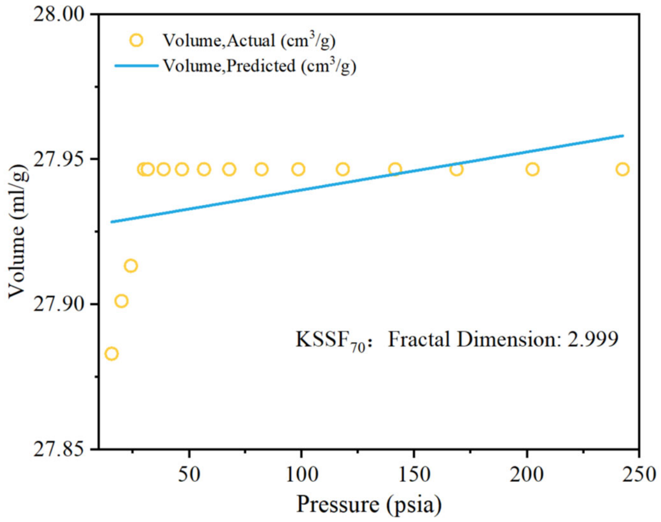

Figure S4 Pore size fractal dimension of $KSSF_{70}$ aerogel obtained from mercury intrusion testing (the theoretical range of the fractal dimension is 2 – 3, where 2 corresponds to an ideally smooth planar structure and 3 corresponds to an extremely complex structure that completely fills space)

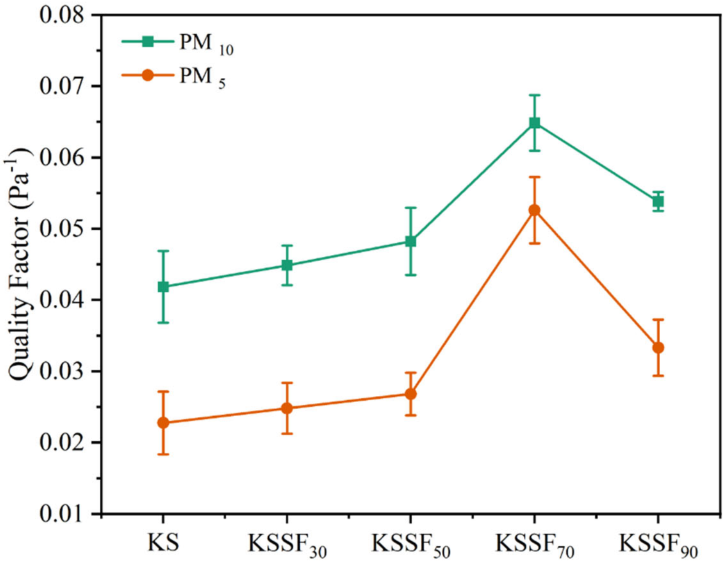

Figure S5 Mass Factor (QF) of KS and KSSF Aerogels of PM_5 and PM_{10}
